# Emergence of a Globally Dominant IncHI1 Plasmid Type Associated with Multiple Drug Resistant Typhoid

**DOI:** 10.1371/journal.pntd.0001245

**Published:** 2011-07-19

**Authors:** Kathryn E. Holt, Minh Duy Phan, Stephen Baker, Pham Thanh Duy, Tran Vu Thieu Nga, Satheesh Nair, A. Keith Turner, Ciara Walsh, Séamus Fanning, Sinéad Farrell-Ward, Shanta Dutta, Sam Kariuki, François-Xavier Weill, Julian Parkhill, Gordon Dougan, John Wain

**Affiliations:** 1 Wellcome Trust Sanger Institute, Hinxton, Cambridge, United Kingdom; 2 Department of Microbiology and Immunology, University of Melbourne, Melbourne, Australia; 3 School of Chemistry and Molecular Biosciences, Australian Infectious Diseases Research Centre, University of Queensland, Brisbane, Queensland, Australia; 4 Oxford University Clinical Research Unit, Wellcome Trust Major Overseas Programme, The Hospital for Tropical Diseases, Ho Chi Minh City, Vietnam; 5 Laboratory of Enteric Pathogens, Health Protection Agency, Colindale, United Kingdom; 6 Food Safety Authority of Ireland, Dublin, Ireland; 7 School of Public Health, Physiotherapy and Population Science, UCD Centre for Food Safety, Veterinary Sciences Centre, University College Dublin, Belfield, Dublin, Ireland; 8 National Institute of Cholera and Enteric Diseases, Kolkata, India; 9 Kenya Medical Research Institute, Nairobi, Kenya; 10 Institut Pasteur, Unité des Bactéries Pathogènes Entériques, Paris, France; Massachusetts General Hospital, United States of America

## Abstract

Typhoid fever, caused by *Salmonella enterica* serovar Typhi (*S*. Typhi), remains a serious global health concern. Since their emergence in the mid-1970s multi-drug resistant (MDR) *S*. Typhi now dominate drug sensitive equivalents in many regions. MDR in *S*. Typhi is almost exclusively conferred by self-transmissible IncHI1 plasmids carrying a suite of antimicrobial resistance genes. We identified over 300 single nucleotide polymorphisms (SNPs) within conserved regions of the IncHI1 plasmid, and genotyped both plasmid and chromosomal SNPs in over 450 *S*. Typhi dating back to 1958. Prior to 1995, a variety of IncHI1 plasmid types were detected in distinct *S*. Typhi haplotypes. Highly similar plasmids were detected in co-circulating *S*. Typhi haplotypes, indicative of plasmid transfer. In contrast, from 1995 onwards, 98% of MDR *S*. Typhi were plasmid sequence type 6 (PST6) and *S*. Typhi haplotype H58, indicating recent global spread of a dominant MDR clone. To investigate whether PST6 conferred a selective advantage compared to other IncHI1 plasmids, we used a phenotyping array to compare the impact of IncHI1 PST6 and PST1 plasmids in a common *S*. Typhi host. The PST6 plasmid conferred the ability to grow in high salt medium (4.7% NaCl), which we demonstrate is due to the presence in PST6 of the Tn*6062* transposon encoding BetU.

## Author Summary

Typhoid fever is caused by the bacterium *Salmonella enterica* serovar Typhi (*S*. Typhi). Treatment relies on antimicrobial drugs, however many *S*. Typhi are multi-drug resistant (MDR), severely compromising treatment options. MDR typhoid is associated with multiple drug resistance genes, which can be transferred between *S*. Typhi and other bacteria via self-transmissible plasmids. We used sequence analysis to identify single nucleotide polymorphisms (SNPs) within these plasmids, and used high-resolution SNP typing to trace the subtypes (termed haplotypes) of both the *S*. Typhi bacteria and their MDR plasmids isolated from more than 450 typhoid patients since 1958. Among isolates collected before 1995, a variety of plasmid haplotypes and *S*. Typhi haplotypes were detected, indicating that MDR typhoid was caused by a diverse range of *S*. Typhi and MDR plasmids. In contrast, 98% of MDR *S*. Typhi samples isolated from 1995 were of the same *S*. Typhi haplotype and plasmid haplotype, indicating that the recent increase in rates of MDR typhoid is due to the global spread of a dominant *S*. Typhi-plasmid combination. We demonstrate this particular plasmid type contains a transposon encoding two transporter genes, enabling its *S*. Typhi host to grow in the presence of high salt concentrations.

## Introduction

Typhoid fever remains a serious public health problem in many developing countries, with highest incidence in parts of Asia (274 per 100,000 person-years) and Africa (50 per 100,000 person-years) [1], [2]. The causative agent is the bacterium *Salmonella enterica* serovar Typhi (*S*. Typhi). While vaccines against *S*. Typhi exist, it is mainly restricted groups such as travellers [3], [4] and individuals enrolled in large vaccine trials [5] who are immunized, and antimicrobial treatment remains central to the control of typhoid fever [3]. However antimicrobial resistant typhoid has been observed for over half a century and is now common in many areas. Chloramphenicol resistant *S*. Typhi was first reported in 1950, shortly after the drug was introduced for treatment of typhoid [6]. By the early 1970s, *S*. Typhi resistant to both chloramphenicol and ampicillin had been observed [7] and multidrug resistant (MDR) *S*. Typhi (defined here as resistance to chloramphenicol, ampicillin and trimethoprim-sulfamethoxazole) emerged soon after [8]. The rate of MDR among *S*. Typhi can fluctuate over time and geographical space, as can the precise combination of drug resistance genes and phenotypes [9], [10]. However in many typhoid endemic areas, an increasing prevalence of MDR *S*. Typhi was observed in the late 1990s [11], [12], [13], and MDR typhoid now predominates in many areas [9], [14] including parts of Asia [15], [16], Africa [17] and the Middle East [18], [19], [20], [21]. MDR *S*. Typhi with reduced susceptibility to fluoroquinolones are increasingly common [9], [15], [16], [22], leaving macrolides or third generation cephalosporins as the only options for therapy [23], [24].

In *S*. Typhi the MDR phenotype is almost exclusively conferred by self-transmissible plasmids of the HI1 incompatibility type (IncHI1) [8], [11], [25], [26], [27], [28], [29], [30], although other plasmids are occasionally reported [31]. In the laboratory, IncHI1 plasmids can transfer between *Enterobacteriaceae* and other Gram-negative bacteria [32] and in nature, IncHI1 plasmids have been detected in pathogenic isolates of *Salmonella enterica* and *Escherichia coli* [33], [34], [35], [36]. However it remains unclear whether the increase in MDR typhoid is due to the exchange of resistance genes among co-circulating *S*. Typhi or to the expansion of MDR *S*. Typhi clones. Efforts have been made to investigate variability within IncHI1 plasmids [29], [33], [37] or their *S*. Typhi hosts [22], [38], [39], [40], [41] but little progress has been made in linking the two together to answer fundamental questions of how MDR typhoid spreads. We recently developed a plasmid multi-locus sequence typing (PMLST) scheme for IncHI1 plasmids, which identified eight distinct IncHI1 plasmid sequence types (PSTs) among *S*. Typhi and *S*. Paratyphi A isolates, including five PSTs found in *S*. Typhi [37]. This pattern was not consistent with a single acquisition of an IncHI1 plasmid in *S*. Typhi followed by divergence into multiple plasmid lineages, rather it indicated that divergent IncHI1 plasmids have entered the *S*. Typhi population on multiple occasions [37]. However the phylogenetic relatedness of the *S*. Typhi hosts was not determined, thus we were unable to estimate how many times plasmids may have been independently acquired.

In this study, we aimed to investigate the relative contribution of plasmid transfer, as opposed to the expansion of plasmid-bearing *S*. Typhi clones, to the emergence of MDR typhoid. We found evidence for plasmid transfer in older *S*. Typhi. However the vast majority of recent MDR typhoid was attributable to a single host-plasmid combination (*S*. Typhi H58-IncHI1 plasmid ST6). We performed further experiments to investigate possible mechanisms for the success of this host-plasmid combination, and identified a transposon in PST6 that confers tolerance to high osmolarity.

## Materials and Methods

### Bacterial isolates and DNA extraction

The bacterial isolates analyzed by SNP assay are summarized in Table 1 and listed in full in Table S1. DNA was extracted using Wizard Genomic DNA purification kits (Promega) according to manufacturer's instructions. Details of the isolates used for competition experiments are also listed in Table S1.

**Table 1:** Summary of 454 *S*. Typhi isolates analyzed in this study.

Region	No. countries	pre-1970s	1970s–1980s	1990s	2000–2007	Total isolates
South & Central America	4	0	6	3	2	11
Central, Southern, East Africa	7	10	3	3	26	42
North Africa	3	11	1	8	5	25
West Africa	11	28	0	6	12	46
East Asia	8	5	8	22	187	222
Indian Subcontinent	3	0	3	1	66	70
Middle East	3	0	0	0	31	31
Europe	5	1	1	2	2	6
Unknown	-	1	0	0	0	1
*Total*	*44*	*56*	*22*	*45*	*331*	*454*

BRD948 is an attenuated Ty2-derived strain (also known as CVD908-*htrA*), which has deletion mutations in *aroC* (t0480), *aroD* (t1231), and *htrA* (t0210) [42]. The growth of BRD948 on LB agar or in LB broth was enabled by supplementation with aromatic amino acid mix (aro mix) to achieve the final concentration of 50 µM L-phenylalanine, 50 µM L-tryptophan, 1 µM para-aminobenzoic acid and 1 µM 2,3-dihydroxybenzoic acid.

### Identification and phylogenetic analysis of IncHI1 SNPs

**Figure 1:**
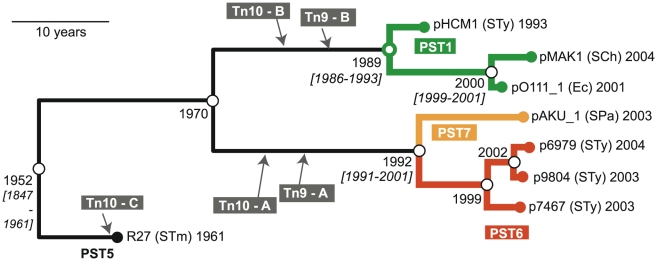
Phylogenetic tree for IncHI1 plasmid sequences. Phylogenetic tree based on 347 SNPs identified among 8 publicly available IncHI1 plasmid sequences (Table 2), constructed using BEAST (with 20 million iterations, 4 replicate runs, exponential clock model). Terminal nodes are labelled with the organism of origin (STy  =  *Salmonella enterica* serovar Typhi, SCh  =  *Salmonella enterica* serovar Choleraesuis, STm  =  *Salmonella enterica* serovar Typhimurium, SPa  =  *Salmonella enterica* serovar Paratyphi A, Ec  =  *E. coli* O111:H-) and date of isolation. Isolation dates were input into the BEAST model in order to estimate divergence dates for internal nodes (open circles, labelled with divergence date estimates; brackets indicate 95% highest posterior density interval). Insertion sites (grey) are based on sequence data and verified (except for pO111_1 and pMAK1) by PCR. Precise insertion sites and PCR primers for verification are given in Tables 3 & 4. Four major plasmid groups, PST1, PST5, PST6, PST7, are coloured as labelled.

Plasmid sequences were downloaded from the European Nucleotide Archive (plasmid details and accessions in Table 2). SNPs between finished plasmid sequences were identified using the *nucmer* and *show-snps* algorithms within the MUMmer 3.1 package [43], via pairwise comparisons with pAKU_1. To identify SNPs in *S*. Typhi PST6 IncHI1 plasmids, 36 bp single-ended Illumina/Solexa sequencing reads from *S*. Typhi isolates E03-9804, ISP-03-07467 and ISP-04-06979 were aligned to the pAKU_1 sequence using Maq [44] and quality filters as described previously [45]. SNPs called in repetitive regions or inserted sequences were excluded from phylogenetic analysis, so that phylogenetic trees were based only on the conserved IncHI1 core regions. This resulted in a total of 347 SNPs, which were analyzed using BEAST [46] to simultaneously infer a phylogenetic tree and divergence dates (using the year of isolation of each plasmid as listed in Table 1, resulting tree in Figure 1). Parameters used were as follows: generalised time reversible model with a Gamma model of site heterogeneity (4 gamma categories); a relaxed molecular clock with uncorrelated exponential rates [46], a coalescent tree prior estimated using a Bayesian skyline model with 10 groups [47], default priors and 20 million iterations.

**Table 2:** IncHI1 plasmid sequences analyzed in this study.

Plasmid	Host	Year of isolation	Plasmid type	Accession	Citation
pHCM1	*S*. Typhi strain CT18	1993	PST1	AL513383	[54]
pAKU_1	*S*. Paratyphi A strain AKU_12601	2003	PST7	AM412236	[33]
R27	*S*. Typhimurium	1961	PST5	AF250878	[65]
pMAK1	*S*. Choleraesuis strain L-2454	2002	PST1	AB366440	-
pO111_1	*E. coli* O111:H- strain 11128	2001	PST1	AP010961	[66]
p9804_1	*S*. Typhi strain E03-9804	2004	PST6	ERA000001	[45]
p7467_1	*S*. Typhi strain ISP-03-07467	2003	PST6	ERA000001	[45]
p6979_1	*S*. Typhi strain ISP-04-06979	2004	PST6	ERA000001	[45]

### SNP typing analysis

**Figure 2:**
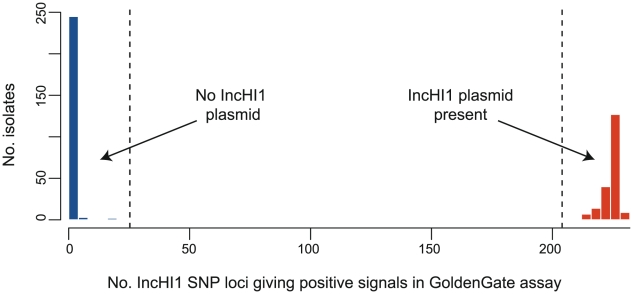
Distribution of IncHI1 loci among *S*. Typhi isolates. X-axis indicates the number of IncHI1 plasmid loci (out of 231 targets) generating a fluorescent signal in the Illumina GoldenGate SNP assay. Isolates clearly fall into two groups: either >90% of IncHI1 target loci were detected, taken to imply presence of an IncHI1 plasmid (red), or <10% of IncHI1 target loci were detected, taken to imply absence of any IncHI1 plasmid (blue).

**Figure 3:**
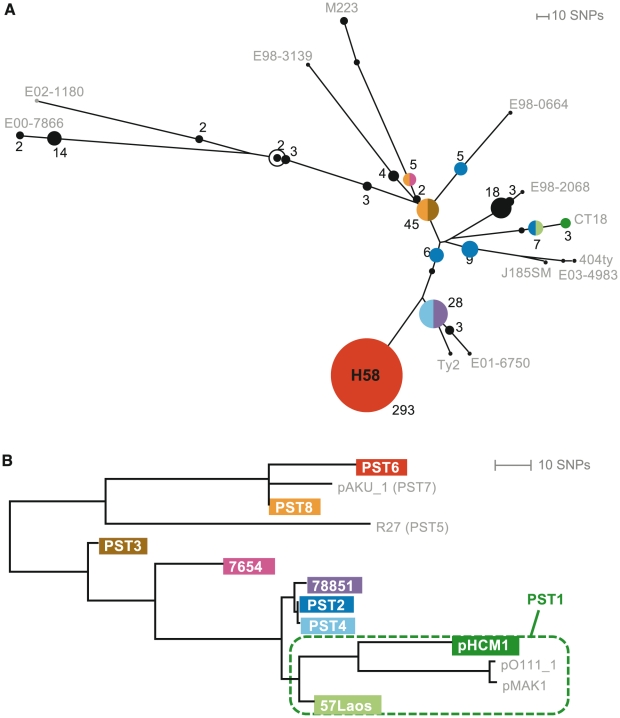
Phylogenetic trees of *S*. Typhi chromosome and IncHI1 plasmid. (A) Phylogenetic tree indicating chromosomal haplotypes of 454 *S*. Typhi isolates determined by SNP typing with the GoldenGate assay. Circles correspond to detected *S*. Typhi haplotypes; node sizes are scaled to the number of isolates detected with that haplotype and labelled with this number. Unfilled circle indicates tree root; reference isolates used to define the *S*. Typhi SNPs are labelled with the isolate name. *S.* Typhi haplotypes in which IncHI1 plasmids were detected (N = 201) are coloured; black circles indicate no IncHI1 plasmids were found among *S*. Typhi of that haplotype; other colours indicate the presence of specific IncHI1 plasmid haplotypes corresponding to the colours in (B). Note that most of the coloured nodes also contain *S*. Typhi isolates with no plasmid, and the colours do not represent the proportion of isolates harbouring the various plasmid types. (B) Phylogenetic tree of IncHI1 plasmids determined by SNP typing with the GoldenGate assay (coloured leaf nodes); grey leaf nodes indicate the position of non-*S*. Typhi plasmids, as determined from plasmid sequence data listed in Table 2.

The chromosomal haplotype of *S*. Typhi isolates was determined based on the SNPs present at 1,485 chromosomal loci identified previously from genome-wide surveys [41], [45] and listed in [22], [39]. IncHI1 plasmid haplotypes were determined using 231 SNPs located in the conserved IncHI1 backbone sequence, listed in Table S2 (note these do not include SNPs specific to pMAK1 or pO111_1 which were not available at the time of assay design, nor any SNPs falling within 10 bp of each other as these cannot be accurately targeted via GoldenGate assay; however additional SNPs identified via plasmid MLST [37] were included, see Table S2). Resistance gene sequences were interrogated using additional oligonucleotide probes, listed in [16]. All loci were interrogated using a GoldenGate (Illumina) custom assay according to the manufacturer's standard protocols, as described previously [16], [22], [39]. SNP calls were generated from raw fluorescence signal data by clustering with a modified version of Illuminus [48] as described previously [22]. The percentage of IncHI1 SNP loci yielding positive signals in the GoldenGate assay clearly divided isolates into two groups, indicating presence of an IncHI plasmid (signals for >90% of IncHI1 loci) or absence of such a plasmid (signals for <10% of IncHI1 loci), see Figure 2. SNP alleles were concatenated to generate two multiple alignments, one for chromosomal SNPs and one for IncHI1 plasmid SNPs. Maximum likelihood phylogenetic trees (Figure 3) were fit to each alignment using RAxML [49] with a GTR+Γ model and 1,000 bootstraps.

### PCR

PCR primers were designed using Primer3 [50] according to the following criteria: melting temperature 56°C, no hairpins or dimers affecting 3′ ends, no cross-dimers between forward and reverse primers. Primer sequences are given in Table 3. PCRs were performed on a TETRA DNA Engine Peltier Thermal Cycler (MJ Research) with a reaction consisting of 1.2 µl of 10X Mango PCR buffer, 1.5 mM MgCl2, 25 µM of each dNTP, 1.25 U Mango *Taq* (Bioline), 0.3 µM of each primer, 1.0 µl DNA template (approx. 100 ng) and nuclease free water in a total reaction volume of 12 µl. Cycling conditions were as follows: 5 min at 94°C, 30 cycles of 15 s at 94°C, 15 s at 58°C, and 60 s at 72°C; final extension of 5 min at 72°C.

**Table 3:** PCR primers for detection of resistance gene insertion sites.

	Forward primer, Reverse primer	Amplicon length in pAKU_1 (bp)	Amplicon length in pHCM1 (bp)
**G**	GATGGAGAAGAGGAGCAACG, TTCGTTCCTGGTCGATTTTC	989	989
**H**	GTGCTGTGGAACACGGTCTA, TCATCAACGCTTCCTGAATG	271	1598
**I**	ACGAAAGGGGAATGTTTCCT, CGAGTGGGAATCCATGGTAG	163	1490
**J**	CAAAATGTTCTTTACGATGCC, CCAGACAGGAAAACGCTCA	2219	none
**K**	CTGTGCCGAGCTAATCAACA, ACGAAAGGGGAATGTTTCCT	1314	none
**L**	TTTTAAATGGCGGAAAATCG, GCCAGTCTTGCCAACGTTAT	none	1872
**M**	GGGCGAAGAAGTTGTCCATA, ATTCGAGCAAAACCATGGAA	none	2195
**N**	CGGGATGAAAAATGATGCTT, GGTCGGTGCCTTTATTGTTG	none	2180
**O**	GCGTACAAAAGGCAGGTTTG, GCTTGATGATGTGGCGAATA	1823	none
**P**	TGGTCGGTGCCTTTATTGTT, GGGCGTCAGAGACTTTGTTC	1899	none
**Q**	TTCGCCCGATATAGTGAAGG, CTAACGCCGAAGAGAACTGG	1923	none

### Plasmid transfer

The transfer of pHCM1 and pSTY7 from respective *E. coli* transconjugants to the attenuated *S.* Typhi BRD948 was performed by cross-streaking onto LB agar supplemented with aro mix and incubating at 37°C overnight. The growth was harvested, resuspended in 2 ml of dH_2_0, plated on MacConkey agar containing streptomycin (1 µg/ml or 5 µg/ml) and chloramphenicol (5 µg/ml or 20 µg/ml) and incubated overnight at 37°C. BRD948 transconjugants were confirmed by antimicrobial susceptibility patterns (disk diffusion) and colony PCR specific for BRD948 background (primers 5939-5′-CGTTCACCTGGCTGGAGTTTG-3′ and5940-5′-CATGCCAGCAGCGCAATCGCG-3′) and pHCM1 or pSTY7 plasmids (Insert1056L- 5′-TAGGGTTTGTGCGGCTTC-3′ and Insert1056R-5′-CCTTCTTGTCGCCTTTGC-3′).

### Competition assays in common host background

The competition between BRD948 (pHCM1) and BRD948 (pSTY7) was started with equal inoculums of roughly 5×10^3^ cfu each in 10 mL of LB broth supplemented with aro mix and chloramphenicol (5 µg/mL). The culture was incubated for 16 hours at 37°C with shaking. Approximately 10^4^ cfu of this culture were then used to inoculate the next passage. The cultures were passaged for a total of 4 days. Samples were collected at time point 0 (at the time of initial inoculation) and after 1, 2, 3 and 4 days of passage, diluted and spread on LB agar supplemented with aro mix. Sixty-four colonies from each sample were randomly picked and tested by PCR to identify their plasmid type (see below). The entire competition assay was performed in triplicate, i.e. beginning with three initial cultures of equal inoculums of the two isolates. The colony PCR was perform using standard condition (see PCR section above) with three primers (DF 5′-CGATTTGTGAAGTTGGGTCA-3′, DR2 5′- CAACCTGGGCAGGTGTAAGT-3′ and DR3 5′- TTCGTTACGTGTTCATTCCA-3′). Expected sizes of PCR products were 511 bp for BRD948 (pHCM1) and 285 bp for BRD948 (pSTY7).

### Competition assays using wildtype isolates

Four individual competitive growth assays were performed using wildtype host-plasmid combinations genotyped using the GoldenGate assay (isolates listed in Table S1); H58-C vs. H1, H58-E1 vs. H1, H58-C-ST6 vs. H1-ST1 and H58-E1-ST6 vs. H1-ST1. Bacterial isolates were recovered from frozen stocks onto Luria-Bertani (LB) media, supplemented with 20 mg/ml of chloramphenicol for isolates with MDR plasmids. Individual colonies were picked and used to inoculate 10 ml of LB broth, which were incubated overnight at 37°C with agitation. Bacterial cells were enumerated the following day by serial dilution and plating. Equivalent quantities of the two competing *S*. Typhi isolates were inoculated into 10 ml of LB broth and were incubated as before (Day 0). The competition assays were conducted by growing the mixed bacteria to stationary phase and then passaging them into 10 ml of LB broth in a 1∶1000 dilution in triplicate over four days. One ml of media containing bacteria from each of the triplicates was stored at −80°C at each time point. DNA was extracted from the frozen samples by boiling for 10 minutes, samples were pelleted, the supernatant was removed and used as template in all of the subsequent competitive real-time PCR reactions (below), which were performed on each template in duplicate.

### Real-time PCR for quantitation of wildtype isolates in competition assays

We performed two individual competitive real-time PCRs (Taqman system) with LNA probes to calculate the proportions of *S.* Typhi H1 vs. *S.* Typhi H58 and *S.* Typhi H58-C vs. *S.* Typhi H58-E1 in aliquots of DNA extracted from broth following competitive growth. These assays were performed to accurately calculate the relative proportion of the isolates in all competitive assays, including those that could not be calculated by plating alone. The haplotype specific primers and probes were designed using Primer Express Software (Applied Biosystems) and manufactured by Sigma-Proligo (Singapore). Primer and probe sequences were as follows (capital letters indicate the position of LNA and the letters in square brackets indicate the SNP position); H58 vs H1 (99 bp amplicon): F(71–83)-CCGAACGCGACGG, R(169-157)-TGCGGCACACGGC and probe 5′-FAM-ccggtAat[G]gtAatGaagc-BHQ1 (*S.* Typhi H1) and 5′-Hex-ccggtAat[A]gtAatGaagc (*S.* Typhi H58); H58-C vs H58-E1 (89 bp amplicon): F(60–75)-ACCCTGCACCGTGACC, R-(148–135)-GCATGATGCCGCCC and probe 5′-FAM-ttcCag[G]ccAtgAcgc –BHQ1 (*S.* Typhi H58-C) and 5′-HEX-ttcCag[A]ccAtgAcgc-BHQ1 (*S.* Typhi H58-E1). PCR amplification were performed using a light cycler (Roche, USA), with hot start Taq polymerase (Qiagen, USA) under the following conditions, 95°C for 15 minutes and 45 cycles of 95°C for 30 seconds, 60°C for 30 seconds and 72°C for 30 seconds. As the primer locations were identical for the internal competitive PCR assay, the efficiency of the PCR was also considered to be identical. Therefore, proportions of isolates at the various time points throughout the assay were calculated by taking the mean of six *Cp* values (each competition assay was performed in triplicate and the PCR was performed in duplicate). The Mean *Cp* values for each competitive assay was converted into a proportion (isolate A) using the following calculation: Proportion isolate A = 1/(2^−ΔCp^ +1), where ΔCp  =  Cp (isolate B) – Cp (isolate A).

### Phenotype microarrays

Phenotype microarrays of osmotic/ionic response (PM 9), pH response (PM 10) and bacterial chemical sensitivity (PM 11 to 20) were performed as described previously by Biolog Inc. (Hayward, California USA) [51]. BRD948 was used as a reference for comparison with BRD948 (pHCM1) or BRD948 (pSTY7) test isolates to identify the phenotypes affected by the presence of IncHI1 plasmid pHCM1 (PST1) or pSTY7 (PST6).

The three isolates were pre-grown on LB (Luria-Bertani) agar plates supplemented with 1X of an aromatic amino acid mix (a 50X aromatic amino acid mix consisted of 50 µM L-phenylalanine, 50 µM L-tryptophan, 1 µM para-aminobenzoic acid and 1 µM 2,3-dihydroxybenzoic acid). Sterile cotton swabs were used to pick colonies and suspend them in 10 ml inoculating media IF-0a (Biolog), the optical density of which was then adjusted to 0.035 absorbance units at 610 nm. A total of 750 µl of this cell suspension was diluted 200 fold into 150 ml inoculating media IF-10 (Biolog), containing 1X aromatic acid mix (1.2X Biolog media, 22 ml of sterile water and 3 ml of 50X aromatic amino acid mix). PM microtitre plates 9–20 were inoculated with 100 µl of the inoculating media cell suspension per well. Microtitre plates were then incubated at 37°C for 48 h in the Omnilog (Biolog Inc) and each well was monitored for colour change (kinetic respiration). Tests were performed in duplicate and the kinetic data was analyzed using the OmniLog PM software set (Biolog Inc). A lower threshold of 80 omnilog units (measured as area under the kinetic response curve) was set, and the phenotypes of each of the three isolates were compared.

### Cloning and growth curves

The fragment of two CDSs within Tn*6062* of pSTY7 (3405 bp) was amplified using two primers IS1056-03 (5′-CAGGCACCGTTTTCTTATTAGAATCTTCGCCACT-3′) and IS1056-04 (5′-TCATTGAACTTTGCTACCCTGA-3′). The pACYC184 fragment (2033 bp) containing its p15A *ori* and chloramphenicol resistant gene (*cmR*) was amplified using pACYC184-01 (5′-AAAATTACGCCCCGCCCTGC-3′) and pACYC184-03 (5′-TAATAAGAAAACGGTGCCTGACTGCGTTAGCA-3′). The two fragments were then fused together by overlapping primer extension PCR (pACYC184-03 and IS1056-03 were two overlapping primers) using pACYC-01 and IS1056-04 primers. All three PCRs above were performed using *PfuUltra* II Fusion HS DNA Polymerase (Agilent, former Stratagene, UK) to achieved highly accurate amplification. The PCRs were set up following the manufacturer's manual with the specific annealing temperature of 58°C and extension time of 45 s for Tn*6062* and pACYC184 fragments or 1.5 min for the fusion fragment. The fused PCR product was re-circularised by T4 ligase (New England BioLabs, UK) to form pACYC184Δ*tet*::Tn*6062* and electroporated into BRD948. The pACYC184 fragment was also re-circularised to form the empty vector pACYC184Δ*tet* and electroporated into BRD948.

Overnight bacterial cultures of BRD948 (pHCM1), BRD948 (pSTY7), BRD948 (pACYCΔ*tet*) and BRD948 (pACYCΔ*tet*::Tn*6062*) were diluted by distilled water to the cell suspension of 0.1 OD600 before 1 µl of the cell suspension was inoculated into 200 µl of 0.8 M NaCl LB broth (supplemented with aro mix) in a well of a 96-well plate. Each isolate was inoculated into six wells (i.e. six biological replicates). The bacteria were grown at 37°C with shaking at 300 rpm and OD_600_ was measured automatically every 15 minutes for 24 hours in the Optima plate reader (BMG Labtech, Germany). Absorbance data were collected and saved in Excel format for graphing.

## Results

### Evolution of MDR IncHI1 plasmids

We compared the DNA sequences of eight ∼200 kbp IncHI1 plasmids isolated from enteric pathogens (Table 2) and identified a conserved IncHI1 core region (>99% identical at the nucleotide level) that included the *tra1* and *tra2* regions encoding conjugal transfer [29], [33], [37], [52]. Subsequently, we identified 347 single nucleotide polymorphisms (SNPs) within these conserved regions, which were used to construct a phylogenetic tree of IncHI1 plasmids and to estimate the divergence dates of internal nodes of this tree based on the known isolation dates for each plasmid [53] (Figure 1). The tree topology is in general agreement with that inferred previously using a plasmid MLST approach [37]. The sequences of the three most recent *S*. Typhi plasmids (isolated 2003–2004) were very closely related and correspond to a previously defined plasmid sequence type (PST) known as PST6 [37] (Figure 1, red). According to our divergence date estimates, the most recent common ancestor (mrca) shared by these three plasmids existed circa 1999 (Figure 1). The PST6 plasmids were also closely related to the PST7 plasmid pAKU_1 from *S.* Paratyphi A (Figure 1, orange), with mrca circa 1992. The plasmids pHCM1, pO111_1 and pMAK1 formed a distinct group corresponding to PST1, with mrca circa 1989 (Figure 1, green). The eighth reference plasmid R27 (PST5) was quite distinct from the others, with an estimated divergence date of 1952 (Figure 1, black).

In addition to the conserved IncHI1 core regions, the plasmids each harbour insertions of drug resistance elements. These include transposons Tn*10* (encoding tetracycline resistance), Tn*9* (encoding chloramphenicol resistance via the *cat* gene (SPAP0067)), *strAB* (SPAP0152-SPAP0153, SPAP0230-SPAP0231; encoding streptomycin resistance), *sul1* and *sul2* (SPAP0132 , SPAP0151; encoding sulfonamide resistance), *dfrA7* (SPAP0133; encoding trimethoprim resistance) and *bla*_TEM-1_ (SPAP0143; encoding ampicillin resistance) [29], [33], [54]. The insertion sites of these elements, confirmed using PCR (Tables 3 & 4), differed between lineages of the IncHI1 phylogenetic tree (Figure 1, grey). All plasmid sequences included Tn*10*, however three different insertion sites were evident (Table 4), suggesting the transposon was acquired by IncHI1 plasmids on at least three separate occasions (Figure 1, grey). Tn*9* was present in all plasmids other than R27, however the insertion site in PST6 and PST7 plasmids differed from that in PST1, suggesting at least two independent acquisitions. It was previously noted that pHCM1 (PST1) and pAKU_1 (PST7) share identical insertions into Tn*9* of a sequence incorporating Tn*21* (including *sul1*, *dfrA7*), *bla*_TEM-1_, *sul2*, and *strAB* [33]; here we found this insertion into Tn*9* was conserved in all PST1 and PST6 plasmid sequences. Together, this composite set of drug resistance elements encodes MDR (resistance to chloramphenicol, ampicillin and trimethoprim-sulfamethoxazole).

**Table 4:** Resistance gene insertion sites in IncHI1 plasmids inferred from a combination of PCR and sequencing.

IncHI1 plasmid sequence type	PST1			PST5	PST6	PST6	PST7	PST8	
**Plasmid or isolate**	pHCM1	pMAK1	pO111_1	R27	p6979	pSTY7	pAKU1	81918	81863, 81424
**Bacterial host**	STy	SCh	Ec	STm	STy	STy	SPa	STy	STy
***Tn10 insertion***	***B***	***B***	***B***	***C***	***A***	***A***	***A***	***A***	***A***
* sequence data*	*B*	*B*	*B*	*C*	*A*	*n/a*	*A*	*n/a*	*n/a*
** N** (Tn*10* - HCM1.247)	+	n/d	n/d	-	-	-	-	-	-
** O** (*tetD* - SPAP0276)	-	n/d	n/d	-	+	+	+	+	+
** P** (SPAP0261 - Tn*10*)	-	n/d	n/d	-	+	+	+	+	+
***Tn9 insertion***	***B***	***B***	***B***	***-***	***A***	***A***	***A***	***-***	***A***
* sequence data*	*B*	*B*	*B*	*-*	*A*	*n/d*	*A*	*n/a*	*n/a*
** J** (*cat* - *trhN*)	-	n/d	n/d	-	+	+	+	-	+
** K** (*mer* - *trhI*)	-	n/d	n/d	-	+	+	+	-	+
** M** (*cat* - HCM1.203)	+	n/d	n/d	-	-	-	-	-	-
L (*insA* - *tetA*)	+	n/d	n/d	-	-	-	-	-	-
***Tn21 into Tn9***	***+***	***+***	***+***	***-***	***+***	***+***	***+***	***-***	***+***
* sequence data*	*+*	*+*	*+*	*-*	*+*	*n/d*	*+*	*n/a*	*n/a*
** H** (*tnpA* - Tn*9*)	+*	n/d	n/d	-	+	+	+	-	+
** I** (*merR* - Tn*9*)	+*	n/d	n/d	-	+	+	+	-	+
***bla/sul/str into Tn21***	***+***	***+***	***+***	***-***	***+***	***+***	***+***	***-***	***+***
* sequence data*	*+*	*+*	*+*	*-*	*+*	*n/d*	*+*	*n/a*	*n/a*
** G** (*strB* – *tniAdelta*)	+	+	+	-	+	n/d	+	-	+
***strAB 2^nd^*** * copy* (SPAP0230- SPAP0231)	***-***	***-***	***-***	***-***	***-***	***-***	***+***	***+***	***+***
* sequence data*	*-*	*-*	*-*	*-*	*-*	*n/d*	*+*	*n/a*	*n/a*
** Q** (*strB* – SPAP0228)	-	n/d	n/d	-	-	-	+	+	+

Summaries of five insertion patterns are shown in bold italics; these are inferred from sequence data where available (italics) and PCR using primers shown in Table 3 (labelled G–Q). STy  =  *Salmonella enterica* serovar Typhi, SCh  =  *Salmonella enterica* serovar Choleraesuis, STm  =  *Salmonella enterica* serovar Typhimurium, SPa  =  *Salmonella enterica* serovar Paratyphi A, Ec  =  *E. coli* O111:H−. + positive PCR result (i.e. successful amplification); - negative PCR result (i.e. no amplification product detected);

*distinct amplicon size for PST1; n/d PCR not done; n/a sequence data not available. “*strAB* 2^nd^” copy refers to the insertion of streptomycin resistance genes *strAB* directly into the plasmid backbone (SPAP0230-SPAP0231), not as part of the *bla/sul/str* element (SPAP0152-SPAP0153).

**Table 5:** Chromosome, plasmid and resistance gene details of drug resistant *S*. Typhi isolated up to 1993*.

Isolate	Year	Country	Chr	Plas	*IS1*	*cat*	*tetA*	*tetC*	*tetD*	*tetR*	*Tn10LR*	*tnpA*	*merAPRT*	*IntI1*	*sul1*	*dhfR*	*dfrA7*	*bla*	*IS26*	*sul2*	*strAB*	*betU*
76–54	1976	Chile	H50	7654	y		y	y	y	y	y											
78–851	1978	Tunisia	H9	78851	y	y	y	y		y	y	y			y							
CT18	1993	Vietnam	H1	PST1	y	y	y	y		y	y	y	y	y		y		y	y	y	y	
76–1406	1976	Indonesia	H42	PST2	y	y	y	y		y	y	y		y	y							
75–2507	1975	India	H55	PST2	y																	
77–302	1977	India	H55	PST2	y	y	y	y		y	y	y		y	y							
77–303	1977	India	H55	PST2	y	y	y	y		y	y	y		y	y							
72–1907	1972	Vietnam	H68	PST2	y	y	y	y		y	y	y		y	y							
72–1258	1972	Mexico	H11	PST3	y	y	y	y	y	y	y	y	y		y							
73–1102	1973	Vietnam	H87	PST4	y	y	y	y		y	y	y			y							
81–863	1981	Peru	H50	PST8	y	y	y	y	y	y	y	y	y		y						y	y
81–424	1981	Peru	H77	PST8	y	y	y	y	y	y	y	y	y	y	y						y	y
81–918	1981	Peru	H77	PST8	y		y	y	y	y	y										y	
57Laos	2000*	Laos	H1	57Laos	y	y	y	y		y	y	y	y	y				y	y	y	y	y
03–4747	2003*	Togo	H42	PST2	y	y	y	y		y	y	y		y	y			y				
04–6845	2004*	Benin	H42	PST2	y	y	y			y	y	y						y				

Chr - *S*. Typhi chromosomal haplotype; Plas - IncHI1 plasmid sequence type;

*- MDR *S*. Typhi isolated after 1993 that were not of the H58 haplotype or PST6 IncHI1 haplotype; y - gene detected in isolate.

### Dissecting the emergence of MDR typhoid

In order to investigate the contribution of distinct IncHI1 plasmid types over time to the emergence of MDR *S*. Typhi, we performed high resolution SNP typing of *S*. Typhi chromosomal and IncHI1 plasmid loci in a global collection of 454 *S*. Typhi, isolated between 1958–2007 (Table 1, Table S1). These isolates include 19 *S*. Typhi isolates sequenced previously [45] and 22 *S*. Typhi isolated from Kenya in 2004–2007 [22]. We also typed eight IncHI1 *S*. Typhi plasmids harboured in *E. coli* transconjugants [29], [37]. SNP typing was performed using the GoldenGate (Illumina) platform to simultaneously assay chromosomal and plasmid SNP loci. We targeted 231 SNPs from the conserved region of the IncHI1 plasmid (Table S2, [37]; note 116 of the 347 identified SNPs were not able to be included in the GoldenGate assay, see Methods) and 119 from resistance genes and associated transposons (see [16]).

Of the 454 *S*. Typhi that we typed, 193 (43%) harboured IncHI1 plasmids, which clustered into nine distinct haplotypes (Figure 3B). As expected, the majority of IncHI1 plasmids harboured multiple resistance genes or elements including Tn*10*, Tn*9*, *strAB*, *sul1*, *sul2*, *dfrA7* and *bla*_TEM-1_. Transposon insertion sites were confirmed for representative plasmids using PCR (Table 4) and agree with the patterns of insertion sites determined by sequencing (Figure 1 & 3B). Thirteen IncHI1 plasmids were identified among *S*. Typhi isolated prior to 1994 (Table 5), including seven of the total nine distinct IncHI1 plasmid haplotypes (Figure 3B).

A total of 26 distinct *S*. Typhi haplotypes were identified by typing of chromosomal SNPs; their phylogenetic relationships are shown in Figure 3A. The PST2 plasmid was detected in three *S*. Typhi haplotypes isolated in Asia between 1972 and 1977 (Table 5), consistent with repeated introduction of closely related IncHI1 plasmids into distinct *S*. Typhi hosts. Similarly, PST8 was present in two *S*. Typhi haplotypes from Peru in 1981 (Table 5) [55], consistent with transfer of the PST8 plasmid among multiple *S*. Typhi haplotypes co-circulating in Peru at this time. Significantly, from 1995 onwards, nearly all IncHI1 plasmids were type PST6 (180/184 plasmids, 98%). Remarkably, there was an exclusive relationship between PST6 plasmids and *S*. Typhi haplogroup H58, with all PST6 plasmids found in *S*. Typhi H58 hosts, and no *S*. Typhi H58 harbouring non-PST6 plasmids (although 35% of *S*. Typhi H58 were non-MDR and plasmid-free). This strongly suggests that the apparent rise in MDR typhoid since the mid-1990s [11], [12], [13] is due to the clonal expansion of H58 *S*. Typhi carrying the MDR PST6 plasmid. This is in contrast to the longer-term situation described above, which showed that in the years following the first emergence of MDR typhoid (1970s–1980s), MDR IncHI1 plasmids had transferred repeatedly into distinct co-circulating *S*. Typhi haplotypes.

**Figure 4:**
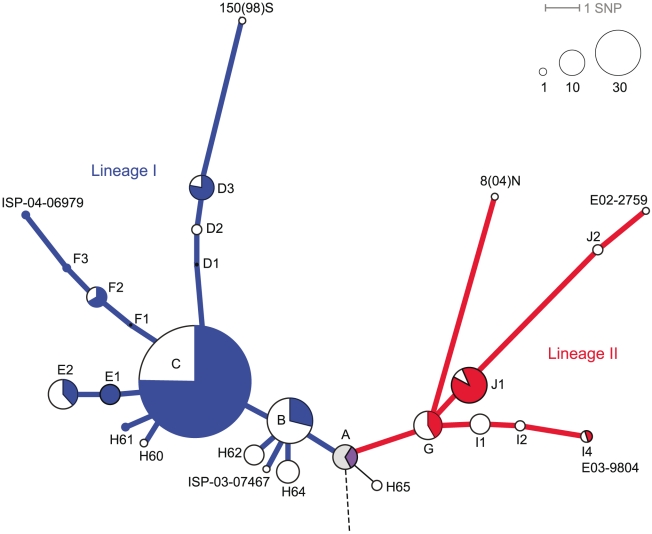
Phylogenetic tree of the H58 haplogroup of *S*. Typhi. Dashed line indicates where this tree joins into the larger phylogenetic tree of *S*. Typhi (shown in Figure 3A). The two major H58 lineages are indicated by colour (blue, lineage I; red, lineage II; purple, common ancestor of both lineages). Nodes are labelled with isolate names (outer nodes representing sequenced isolates; see [45]), haplotype (H followed by number, as defined in [41]) or letters indicating nodes resolved by SNP typing. Node sizes indicate the relative frequency of each haplotype within the study collection of 269 H58 *S*. Typhi isolates, according to the scale provided. The proportion of isolates in each node carrying the PST6 plasmid and IS*1* (solid colour), IS*1* only (white) or neither (grey) is indicated by shading.

The clonal expansion of H58 *S*. Typhi has been documented previously [22], [41], however the role of the PST6 plasmid has not been investigated. Among our collection, the oldest *S*. Typhi H58 isolate dates back to 1995 and carries the PST6 plasmid. To ascertain whether the common ancestor of *S*. Typhi H58 might have carried the PST6 plasmid, the phylogenetic structure among our 293 *S*. Typhi H58 isolates was resolved using 45 of the assayed SNP loci that differentiate within the H58 haplogroup (Figure 4). These SNPs divided the isolates into 24 distinct H58 haplotypes, with the majority (N = 270) in 13 haplotypes (Figure 4). Most of the H58 haplotypes (N = 14), including the ancestral haplotype A, included isolates harbouring the PST6 plasmid (Figure 4). We have previously sequenced the genomes of 19 *S*. Typhi, including seven isolates from the H58 haplogroup [45], and observed the insertion of an IS*1* transposase between protein coding sequences STY3618 and STY3619 within all sequenced H58 *S*. Typhi genomes. This transposase was identical at the nucleotide level to the IS*1* sequences within Tn*9* in IncHI1 plasmids pHCM1 and pAKU_1, and shared a common insertion site in all seven *S*. Typhi H58 chromosomes sequenced [45]. In the present study, our SNP assays included a probe targeting sequences within the IS*1* gene (SPAP0007). Nearly all of the *S*. Typhi H58 isolates gave positive signals for this IS*1* target (Figure 4; coloured or white), with the sole exception of six isolates belonging to the H58 ancestral haplotype A (Figure 4, grey), which also included three isolates that carried the PST6 plasmid and tested positive for IS*1* (Figure 4, purple). This suggests that the PST6 plasmid was likely acquired by the most recent common ancestor of *S*. Typhi H58 (Figure 4, haplotype A), followed by transposition of IS*1* into the *S*. Typhi chromosome prior to divergence into subtypes of H58. Thus the dominance of PST6 over other MDR IncHI1 plasmids (noted here and previously [37]) and the dominance of H58 over other *S*. Typhi haplotypes (noted here and previously [22], [41]) appears to be the result of a trans-continental clonal expansion of MDR *S*. Typhi H58 carrying the PST6 plasmid.

### Possible selective advantages of IncHI1 PST6

These results indicate that the recent global spread of MDR typhoid is attributable to the emergence of a single plasmid-host combination (H58-PST6). We were able to transfer the PST6 plasmid pSTY7 from *S*. Typhi to *E. coli* [29] and back to *S*. Typhi (data not shown), confirming that the PST6 plasmid retains the ability to transfer between bacteria via conjugation, yet we found no evidence of PST6 transfer in natural *S*. Typhi populations (above). This raises the question of why this particular plasmid-host association has been so successful and exclusive.

**Figure 5:**
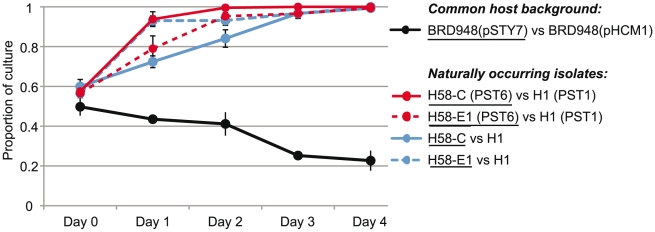
Competitive growth assays for *S*. Typhi H58 and H1 with and without IncHI1 plasmids. The dynamics of five competitive growth assays conducted over four days of sequential sub-culture. Black line indicates competition in a common host background (attenuated laboratory strain *S*. Typhi BRD948; haplotype H10); the proportion of PST1- and PST6-bearing bacteria at each time point was calculated by streaking an aliquot of the sample onto agar plates and testing random colonies using a PCR that differentiates PST1 and PST6. Coloured lines indicate competition between wildtype *S*. Typhi isolates as specified in the legend (see Table S1 for isolate names); the proportion of H58 and H1 chromosomes at each time point was calculated by quantifying the relative abundance of two alleles at a SNP locus that differs between H58 and H1 *S*. Typhi using quantitative PCR. For all assays, experiments were replicated at least three times; data points represent the mean proportion of culture corresponding to the isolate underlined in the legend; error bars show the standard deviation of this proportion.

To investigate whether PST6 could confer any selective advantage over other IncHI1 plasmids harbouring similar antimicrobial resistance genes, representative PST6 (pSTY7) and PST1 (pHCM1) IncHI1 plasmids from Vietnamese *S*. Typhi were introduced into a common *S*. Typhi BRD948 host, derived from *S*. Typhi Ty2 (haplotype H10). The PST1 plasmid pHCM1 was chosen for comparison since its complete sequence is available [54] and it was previously observed to be common in MDR *S*. Typhi in Vietnam in the early 1990s, just prior to the emergence of PST6 in *S*. Typhi in Vietnam and elsewhere [29]. BRD948 (pHCM1) grew to three times the number of cfu compared to BRD948 (pSTY7) after 4 days of mixed growth in LB broth (Figure 5, black). We therefore hypothesized that the advantage conferred by PST6 plasmids, if any, might be related to specific environmental conditions or to plasmid-host compatibility. To test the latter, we compared the growth of wildtype PST1-bearing *S*. Typhi H1 and PST6-bearing *S*. Typhi H58 isolated from typhoid patients in Vietnam and Pakistan and genotyped using the GoldenGate assay (listed in Table S1). The two PST6-bearing *S*. Typhi H58 isolates tested were both able to out compete the PST1-bearing H1 isolate, so that *S*. Typhi H1 was barely detectable after four days of competitive growth (Figure 5, red). However plasmid-free *S.* Typhi H58 isolates were also able to outcompete a plasmid-free *S*. Typhi H1 isolate (Figure 5, blue), thus we cannot confirm the plasmid plays a role in the competitive advantage of H58-PST6 *S*. Typhi over and above that of the H58 chromosomal haplotype.

**Figure 6:**
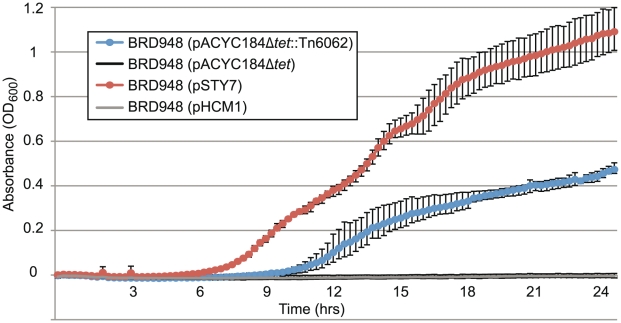
The effect of Tn*6062* on osmotolerance in *S.* Typhi BRD948. Growth curves for *S*. Typhi isolates in 0.8 M NaCl LB broth. Error bars indicate range of maximum and minimum values.

To screen for conditions under which PST6 plasmids confer an advantage compared to PST1 plasmids, we used Biolog phenotyping arrays to compare the growth of plasmid-free *S*. Typhi BRD948 to BRD948 (pHCM1) and BRD948 (pSTY7) under a wide variety of conditions including various pH levels and osmotic/ionic strengths, and a wide variety of antibiotics and chemicals [51]. As expected, both IncHI1 plasmids conferred enhanced growth in the presence of a wide range of antibiotics including amoxicillin, azlocillin, oxacillin, penicillin G, phenethicillin, chloramphenicol, streptomycin, gentamicin, tetracyclines and trimethoprim (Table S3). BRD948 (pHCM1) displayed some minor growth advantages in the presence of additional antimicrobials, however none of these reached clinically relevant levels (Table S3). The only conditions under which BRD948 (pSTY7) grew better than BRD948 and BRD948 (pHCM1) was under high osmotic stress (3-5% NaCl or 6% KCl) (Table S3). We confirmed this phenotype by inoculating each isolate into high salt concentration media (0.8 M NaCl LB broth, approx. 4.7% NaCl); only the PST6-bearing isolate BRD948 (pSTY7) was able to grow under these conditions (Figure 6, red and grey).

We hypothesised that the osmotolerant properties of PST6 plasmids may be explained by the presence of two putative transporters encoded within a composite transposon, Tn*6062* (SPAP0100, SPAP0105, SPAP0106, SPAP0110; this transposon was referred to as Ins*1056* in [37]). Tn*6062* was present in all PST6 plasmids, the novel subtype of PST1 (57Laos) and two of the three PST8 plasmids, but absent from all other isolates (detected via two Tn*6062*-specific probes included in our SNP typing assay). To determine if Tn*6062* was responsible for the osmotolerant phenotype of BRD948 (pSTY7), the two putative transporter genes from Tn*6062* (SPAP0105 and SPAP0106) were inserted into the plasmid vector pAYCY184 and we assessed their effect on *S*. Typhi BRD948 in high salt concentration medium (0.8 M NaCl LB broth, approx. 4.7% NaCl). BRD948 (pAYCY184-Tn*6062*) was able to grow at a slightly lower rate than BRD948 (pSTY7) (Figure 6, blue), while BRD948 carrying the empty pAYCY184 vector was unable to grow (Figure 6, black). Therefore the transposon Tn*6062* carried by the PST6 IncHI1 plasmids confers an osmotolerant phenotype on its *S*. Typhi host.

## Discussion

Our analysis of IncHI1 plasmid sequences indicates that plasmids responsible for the MDR phenotype in *S*. Typhi are closely related to those associated with MDR in other enteric pathogens including *S*. Paratyphi A, *S*. Choleraesuis and enterohaemorrhagic *E. coli* O111:H- (Figure 1, Table 2). These plasmids share a recent common ancestor approximately six decades old and have evolved into several distinct lineages via accumulation of point mutations, followed by acquisition of resistance elements and further point mutation (Figure 1). Simultaneous SNP typing of plasmid and host enabled us to differentiate between the clonal expansion of MDR *S*. Typhi, and independent acquisitions of related MDR plasmids by distinct *S*. Typhi hosts. Evidence for the latter includes the detection of PST2 and PST8 plasmids in co-circulating *S*. Typhi isolates of distinct haplotypes in the 1970s and 1980s (Table 5). This indicates that the emergence of MDR typhoid during this period was in part due to transfer of IncHI1 plasmids within local *S*. Typhi populations. One of the PST2-*S*. Typhi combinations (chromosomal haplotype H42) was later detected among two isolates from Africa in 2003–2004, suggesting that an individual IncHI1 plasmid may be able to persist in a single host haplotype for decades (Table 5). In stark contrast, all 193 PST6 plasmids were observed in *S*. Typhi of the H58 haplotype and virtually all MDR *S*. Typhi observed after 1995 belonged to the same PST6-H58 clone, indicative of global spread of MDR typhoid via clonal expansion. Since humans are the only known reservoir for *S*. Typhi [56], it is likely that trans-continental spread of this clone depends on international travel or migration. If this is the case it will be particularly difficult to control since *S*. Typhi can be transmitted by asymptomatic carriers [57], [58], who are usually unaware of their status and are difficult to detect [59], [60].

Our data suggest that the PST6 plasmid was acquired by the most recent common ancestor of *S*. Typhi H58 (Figure 4), implying that the expansion of *S*. Typhi H58 did not begin until after acquisition of the plasmid. To our knowledge, the oldest confirmed *S*. Typhi H58 isolate is 9105928K [41], which was isolated in India in 1991 and is MDR (Mia Torpdhal, personal communication). This suggests that the initial expansion of *S*. Typhi H58 may have been associated with the acquisition of the PST6 plasmid, implying a selective advantage over and above that of MDR, which was also conferred by other IncHI1 plasmid types circulating in *S*. Typhi in the 1990s. The only growth advantage we detected for PST6 plasmids via our phenotype screen was that of osmotolerance, which we showed to be conferred by the Tn*6062* transposon carried by PST6 plasmids. The transposon Tn*6062* includes betU (SPAP0106), which encodes a betaine uptake system capable of transporting glycine betaine and proline betaine [61]. It was first described in *E. coli* isolates causing pyelonephritis (ascending urinary tract infection) and is believed to be an osmoregulator, allowing *E. coli* to survive the high osmolarity and urea content in urine [61]. However the gene is distributed among *E. coli* with a range of pathogenic phenotypes, so its osmoprotectant properties may be useful in other environmental contexts [62]. It is possible that enhanced osmotolerance may enhance survival of *S*. Typhi in specific niches within the human body, including the gut, gall bladder, urinary tract or liver. It is also possible that the ability to grow in the presence of high salt concentrations might enable *S*. Typhi to continue replicating in certain environments outside the host, which may lower the infectious dose or enhance the possibility of transmission by increasing the level of *S*. Typhi contamination in certain environments. This may have contributed to the selection of PST6 over other IncHI1 plasmids previously circulating among *S*. Typhi and the initial clonal expansion of H58 *S*. Typhi, however questions remain as to why the PST6 plasmid has not been detected among non-H58 *S*. Typhi. The PST6 plasmid appears to have been lost from H58 *S*. Typhi in some areas where the recommended treatment of typhoid was switched to fluoroquinolones, including Nepal and Vietnam [39], [63], [64], while it has been maintained in areas such as Kenya where chloramphenicol is still commonly used to treat typhoid [17], [22]. This confirms that antimicrobial use exerts a strong selective pressure for maintenance of the IncHI1 plasmid among *S*. Typhi and indicates that in the absence of such pressure any additional advantages conferred, including the increased osmotolerance described above, is not enough to maintain the PST6 plasmid indefinitely.

## Supporting Information

Table S1 **Bacterial isolates analyzed in this study.**
(XLS)

Table S2 **IncHI1 SNP loci targeted in this study.**
(XLS)

Table S3 **Biolog phenotype array results.**
(XLS)
